# GPrank: an R package for detecting dynamic elements from genome-wide time series

**DOI:** 10.1186/s12859-018-2370-4

**Published:** 2018-10-04

**Authors:** Hande Topa, Antti Honkela

**Affiliations:** 1 Institute for Molecular Medicine Finland FIMM, University of Helsinki, Helsinki, 00014 Finland; 2 Helsinki Institute for Information Technology HIIT, Department of Computer Science, Aalto University, Espoo, 00076 Finland; 3 Helsinki Institute for Information Technology HIIT, Department of Mathematics and Statistics, University of Helsinki, Helsinki, 00014 Finland; 4 Department of Public Health, University of Helsinki, Helsinki, 00014 Finland

**Keywords:** Gaussian process, High-throughput sequencing, Time series, Ranking, Bayes factor, Visualization, R

## Abstract

**Background:**

Genome-wide high-throughput sequencing (HTS) time series experiments are a powerful tool for monitoring various genomic elements over time. They can be used to monitor, for example, gene or transcript expression with RNA sequencing (RNA-seq), DNA methylation levels with bisulfite sequencing (BS-seq), or abundances of genetic variants in populations with pooled sequencing (Pool-seq). However, because of high experimental costs, the time series data sets often consist of a very limited number of time points with very few or no biological replicates, posing challenges in the data analysis.

**Results:**

Here we present the *GPrank* R package for modelling genome-wide time series by incorporating variance information obtained during pre-processing of the HTS data using probabilistic quantification methods or from a beta-binomial model using sequencing depth. *GPrank* is well-suited for analysing both short and irregularly sampled time series. It is based on modelling each time series by two Gaussian process (GP) models, namely, time-dependent and time-independent GP models, and comparing the evidence provided by data under two models by computing their Bayes factor (BF). Genomic elements are then ranked by their BFs, and temporally most dynamic elements can be identified.

**Conclusions:**

Incorporating the variance information helps *GPrank* avoid false positives without compromising computational efficiency. Fitted models can be easily further explored in a browser. Detection and visualisation of temporally most active dynamic elements in the genome can provide a good starting point for further downstream analyses for increasing our understanding of the studied processes.

## Background

Advances in high-throughput sequencing (HTS) technologies have facilitated carrying out genome-wide time series experiments which contain more information on the dynamics of biological processes than static experiments do. With these experiments, thousands or millions of genomic elements can be simultaneously measured at a number of time points, allowing us to study the changes in their abundances over time, and hence to model their responses to various external stimuli such as a drug treatment or a change in environment. Furthermore, detection of temporally most active elements in the genomes, transcriptomes, or epigenomes of the organisms can lead to a subset of genetic elements which are potentially biologically more relevant to the studied process than those which stay unchanged. This subset of genetic elements can then form a basis for further downstream analyses to elucidate and validate their functions in the studied processes.

On the other hand, despite the huge potential of HTS time series experiments, analysis of the currently available HTS time series data sets is complicated due to various factors depending on the experimental design and the properties of the HTS platforms used. First of all, these time series often consist of small number of time points which are irregularly sampled, making the estimation of the underlying temporal function challenging, and they have too few biological replicates for accurate estimation of biological variance. Moreover, the properties of the HTS platforms such as short read lengths and varying sequencing depth levels lead to uncertain quantification of the genetic elements.

Taking these characteristics of the data as well as the sources of uncertainty into account in the downstream analyses such as differential expression (DE) analyses is very important for avoiding large numbers of false positives or false negatives. This becomes especially important in large-scale studies like genome-wide experiments, as finding differentially expressed genes among tens of thousands of genes requires robust statistical methods which can differentiate true changes from changes occurring due to noise.

Detection of differentially expressed genes from HTS time series is handled in different ways by different methods. For example, some methods treat time points as independent factors and apply statistical hypothesis testing to detect statistically significant changes in gene expression between different time points. For example, edgeR [1], DESeq2 [2], limma-voom [3], next maSigPro [4] are commonly used methods to detect DE between different time points by modelling RNA-seq read counts with generalized linear models which treat the time points as unordered factors.

Recently, methods which take into account the temporal correlation between observations in RNA-seq experiments have been developed by using hidden Markov models (HMMs) [5], cubic spline regression [6], and Gaussian process (GP) regression [7–12].

Similarly, in population genetics, several methods taking into account the temporal correlations between allele frequencies in successive generations have been developed by using HMMs based on the Wright–Fisher model [13, 14], which usually assume a large population size and a long time span. Recently developed CLEAR method [15] improves the HMM models by making them applicable to data sets obtained from small populations such as Pool-seq time series in evolve and resequence (E &R) [16] studies.

GPs provide a powerful technique for modelling sparse time series which are encountered frequently in genomic studies where the number of replication and the length of time series are limited by the experiment budget. However, most of the existing methods employing GPs for HTS time series modelling are either not available as software, or the existing software such as DyNB [10] has been implemented in Matlab, limiting the public accessibility of the software.

In our earlier papers [17, 18], we applied GP modelling to multiple short time series in RNA-seq and Pool-seq applications, and identified temporally most active genomic elements by using Bayes factors (BFs), which measure the evidence provided by the data for being generated by a temporally-changing model rather than a constant model. GP models were further strengthened against model over-fitting by incorporating uncertainty information obtained from data pre-processing stages into the GP models.

In this paper we present *GPrank*, a user-friendly R [19] package which provides a unified interface to GP modelling of different types of genomic time series. *GPrank* builds upon the *gptk* package by [8], and introduces a clean interface for incorporating the pre-processing variances and includes improvements in the optimization.

## Implementation

**Fig. 1:**
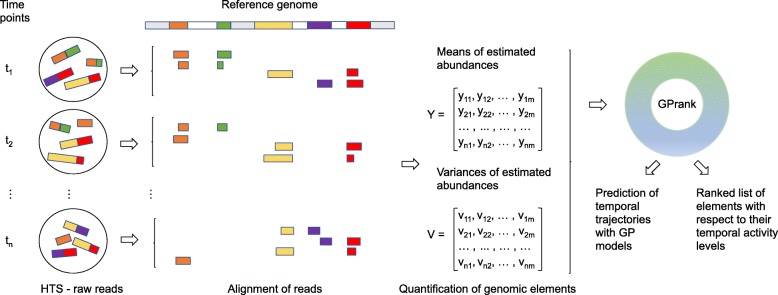
*GPrank* analysis workflow. Application of HTS at *n* time points produces millions of short reads. These short reads are then aligned to a reference sequence (e.g., genome or transcriptome) and then the abundances of the genetic elements are estimated. *GPrank* requires two matrices as input data: a matrix *Y* which contains the mean abundances of *m* genetic elements estimated at *n* time points and a matrix *V* which contains the corresponding variances for the estimated abundances

Figure 1 illustrates a typical workflow for the *GPrank* analysis. *GPrank* requires that the HTS time series data have gone through the pre-processing stages, and the abundances of the genomic elements have been estimated by probabilistic methods, leading to two matrices, one of which contains the estimated mean abundances of genomic elements and the other contains corresponding variance levels. *GPrank* then utilises this information in the GP models of time series.

Depending on the application, different methods can be used to obtain the mean and variance information which is required for *GPrank*. For example, transcript isoform quantification can be handled by methods like RSEM [20], MISO [21], MMSEQ [22], BitSeq [23] or Kallisto [24] in RNA-seq applications, and allele frequencies can be estimated by methods like CRISP [25] and PoPoolation [26] in Pool-seq applications.

Once the genomic elements have been quantified with some degree of confidence at the given time points, *GPrank* can be used to model the time series by utilising the obtained mean and variance information. The method underlying the *GPrank* package works such that each time series is modelled by GP regression with two different models, namely, time-dependent and time-independent models. The time-dependent model assumes that the observations at different time points are correlated with each other. This temporal correlation is captured by using a squared exponential, i.e., radial basis function (RBF) kernel [27] which has two free hyper-parameters: length-scale *ℓ* and the signal variance $\sigma ^{2}_{f}$. The observation noise is assumed to be normally distributed with zero-mean and variance $\sigma ^{2}_{n}+v_{i}$ where $\sigma ^{2}_{n}$ is a free hyper-parameter denoting the global noise variance, and *v*_*i*_ is the fixed variance obtained from pre-processing. The time-independent model, i.e. the null model, assumes that the observations are independently distributed around a constant function with the observation noise having the same distribution as in the time-dependent model.

Free hyper-parameters are then estimated by maximizing the marginal likelihoods, and BFs are computed by the ratio of the maximum marginal likelihoods under the two alternative models. When maximizing the marginal likelihood, the minimum sampling distance is introduced as a lower bound to the length-scale of the RBF kernel in order to satisfy the compatibility with the sampling regime of the time series [28], and the fixed variances serve as a lower bound for the global noise variance $\sigma ^{2}_{n}$. Introducing these bounds helps to guarantee that the marginal likelihood surface is well-behaved, and hence alleviates over-fitting problems which can lead to inflated BFs [28].

Higher BF corresponds to higher support for the time-dependent model. According to [29], ln(BF)>3 indicates strong evidence in favour of the time-dependent model. This cut-off roughly corresponds to 95% posterior probability for the time-dependent model when equal prior probabilities are assumed for both models, which would directly translate to 5% false discovery rate in multiple testing. However, different cut-off values can still be specified depending on the study and the expertise of the researcher. BFs do not have a uniform distribution under the null like p-values, and hence they do not require multiple testing correction.

For the technical details of GP models, we refer to [27], and for performance evaluation of the GP models with and without variance incorporation, we refer to our earlier papers [17] and [18] where it was shown that the variance incorporation in the GP models can yield a higher precision by alleviating the over-fitting problems and helping to reduce the number of false discoveries. This is especially an important issue in genome-wide studies where interesting genomic elements usually account for a very small fraction of the whole data.

**Fig. 2:**
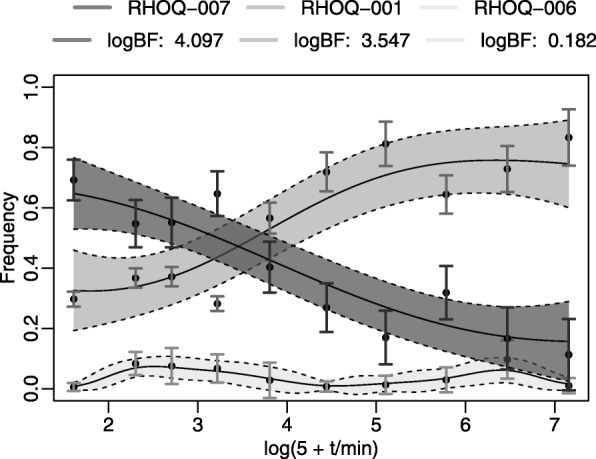
An illustrative example of the fitted GP models for three transcripts originated from RHOQ gene. GP models and the observations for each transcript are differentiated by different shades of gray. Relative frequencies of the transcripts are given on the y-axis and the transformed time points are given on the x-axis. Error bars denote 2 standard deviations which were obtained from pre-processing and the shaded areas denote 2 standard deviations confidence region for the fitted GP models. Higher log-BF indicates more evidence for a time-dependent model. The time series RNA-seq data have been provided in [33] and also analysed in [18] where log(5+**t**) transformation was applied to the time points **t**=[0,5,10,20,40,80,160,320,640,1280] prior to GP modelling

As reference to our earlier papers, *GPrank* directly supports incorporating uncertainty information from a beta-binomial model of the allele frequencies depending on the number of allele counts and the sequencing depth in Pool-seq experiments [17], and the uncertainty on the gene and transcript expression levels estimated by BitSeq [23] from RNA-seq reads [18]. Figure 2 shows an example of the fitted GP models for three transcripts from [18] whose relative expression levels have different uncertainties at different time points. Users can also implement their own variance estimation methods depending on the nature of the data which may be in discrete or continuous values, or in ratios, and may have undergone different data acquisition and pre-processing procedures.

*GPrank* allows to visualize GP profiles of the time series and it supports exporting the results to *tigreBrowser* [30, 31] for further exploration. Genomic elements can then be filtered by using their BFs or any other criteria specified by the user. Similar filtering approaches have been employed, for example, in [32, 33].

**Fig. 3:**
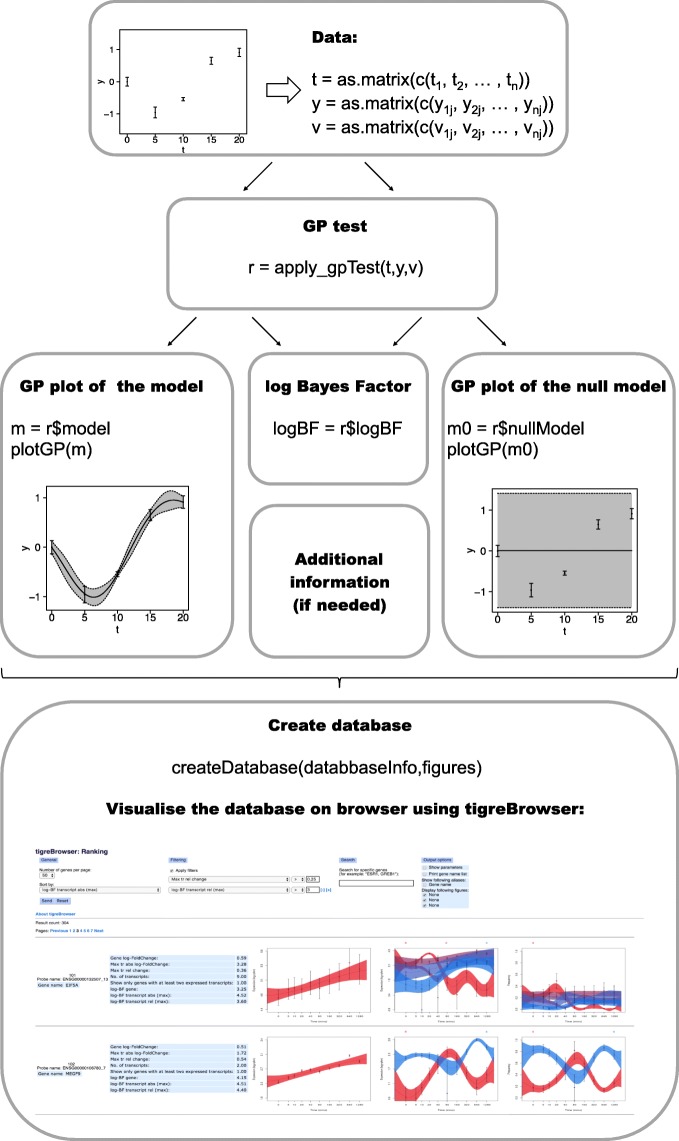
Schema displaying the use of *GPrank* functions. Time series data of each genetic element in the data set are represented by three one-column matrices: **t**: time points; **y**: estimated abundances at the corresponding time points; **v**: variances of the estimated abundances at the corresponding time points. These matrices are then given as input to the apply_gpTest() function. apply_gpTest() function optimises the time-dependent (*m*) and time-independent (*m*0) models and computes the natural logarithm of BF. The kernel structures are specified by default as (“rbf”, “white”, “fixedvariance”) in the time-dependent model, and as (“white”, “fixedvariance”) in the time-independent model. Fitted GP models can be plotted by plotGP() function. Finally, an SQL database can be created with createDatabase() function, allowing inclusion of the figures and additional information (e.g., BFs, fold changes) for visualisation, ranking, and filtering purposes. The created database can be viewed using *tigreBrowser* [30]

The main functions of *GPrank* have been briefly described in Fig. 3. More detailed explanations about the usage of the functions and further examples can also be found in the vignette inside the package.

## Results and discussion

Existing software packages which perform DE analysis from RNA-seq time course data have recently been evaluated in a comparison study [34]. Each of these packages employs its own strategy for normalization and variance modelling for a particular data type, and hence fails to be flexible enough to be used in wider range of applications. Although *GPrank* includes examples on mean and variance modelling in RNA-seq and Pool-seq data, it is also flexible to be used with any kind of HTS data by allowing users to first apply their own method to estimate the mean and variance information by choosing the most suitable method based on the characteristics of their data and their expertise.

Our package can then be used to fit GP models by taking into account the provided variances on the estimated quantities, and ranks the genomic elements according to their temporal activity levels. By doing this, we aim at obtaining the most plausible ranking under the limitations and characteristics of the data set. This makes our method robust against the uncertainty in the data and proves useful to avoid high numbers of false positives.

It is also worth mentioning that our method currently models time series of each genomic element independently of the time series of other genomic elements in the data set, which might lead to information loss. Multi-locus analyses which also account for the correlations between different genetic elements could be an interesting venue for further software development. For example, multi-locus approaches have recently been employed for modelling allele frequency changes in evolutionary processes by [35, 36]. However, more research should be done to make these methods computationally efficient and practical to use in real-life problems.

## Conclusions

Here we presented the *GPrank* package which can be used to identify dynamic elements which show significant and consistent temporal changes among many candidate elements. The method is based on GP modelling of multiple short time series by utilizing the available variance information on the observations. Variance incorporation strengthens the models against over-fitting and it proves useful in needle-in-a-haystack-like problems in which the number of interesting elements is very small in comparison to the number of all candidate elements in the whole genome.

Allowing for visualization and filtering, we believe that our package will be useful for researchers to gain insight into the temporal structures of the time series involved in their experiments and to form a basis for further downstream analyses.

Our method can be applied not only in RNA-seq time series, but also in other genome-wide time series such as DNA methylation time series in epigenomics studies and Pool-seq time series in population genetics studies.

## Availability and requirements

**Project name:***GPrank*,

**Project home page:**https://CRAN.R-project.org/package=GPrank,

**Operating system(s):** Windows, Linux, MacOS,

**Programming language:** R,

**Other requirements:** Python,

**License:** MIT License,

**Any restrictions to use by non-academics:** No

## Abbreviations

BF: Bayes factor
BS-seq: Bisulfite sequencing
DE: Differential expression
E &R: Evolve and resequence
GP: Gaussian process
HMM: Hidden Markov model
HTS: High-throughput sequencing
Pool-seq: Pooled sequencing
RBF: Radial basis function
RNA-seq: RNA sequencing

## References

[1] Robinson MD, McCarthy DJ, Smyth GK (2010). edgeR: a Bioconductor package for differential expression analysis of digital gene expression data. Bioinformatics.

[2] Love MI, Huber W, Anders S (2014). Moderated estimation of fold change and dispersion for RNA-seq data with DESeq2. Genome Biol.

[3] Ritchie ME, Phipson B, Wu D, Hu Y, Law CW, Shi W, Smyth GK (2015). limma powers differential expression analysis for RNA-sequencing and microarray studies. Nucleic Acids Res.

[4] Nueda MJ, Tarazona S, Conesa A (2014). Next maSigPro: updating maSigPro bioconductor package for RNA-seq time series. Bioinformatics.

[5] Leng N, Li Y, McIntosh BE, Nguyen BK, Duffin B, Tian S, Thomson JA, Dewey CN, Stewart R, Kendziorski C (2015). EBSeq-HMM: a Bayesian approach for identifying gene-expression changes in ordered RNA-seq experiments. Bioinformatics.

[6] Michna A, Braselmann H, Selmansberger M, Dietz A, Hess J, Gomolka M, Hornhardt S, Blüthgen N, Zitzelsberger H, Unger K (2016). Natural cubic spline regression modeling followed by dynamic network reconstruction for the identification of radiation-sensitivity gene association networks from time-course transcriptome data. PLoS ONE.

[7] Stegle O, Denby KJ, Cooke EJ, Wild DL, Ghahramani Z, Borgwardt KM (2010). A robust Bayesian two-sample test for detecting intervals of differential gene expression in microarray time series. J Comput Biol.

[8] Kalaitzis AA, Lawrence ND (2011). A simple approach to ranking differentially expressed gene expression time courses through Gaussian process regression. BMC Bioinformatics.

[9] Hensman J, Lawrence ND, Rattray M (2013). Hierarchical Bayesian modelling of gene expression time series across irregularly sampled replicates and clusters. BMC Bioinformatics.

[10] Äijö T, Butty V, Chen Z, Salo V, Tripathi S, Burge CB, Lahesmaa R, Lähdesmäki H. (2014). Methods for time series analysis of RNA-seq data with application to human Th17 cell differentiation. Bioinformatics.

[11] Heinonen M, Guipaud O, Milliat F, Buard V, Micheau B, Tarlet G, Benderitter M, Zehraoui F, d’Alché-Buc F (2015). Detecting time periods of differential gene expression using Gaussian processes: an application to endothelial cells exposed to radiotherapy dose fraction. Bioinformatics.

[12] Yang J, Penfold CA, Grant MR, Rattray M (2016). Inferring the perturbation time from biological time course data. Bioinformatics.

[13] Bollback JP, York TL, Nielsen R (2008). Estimation of 2 *N*_*e*_s from temporal allele frequency data. Genetics.

[14] Feder AF, Kryazhimskiy S, Plotkin JB (2014). Identifying signatures of selection in genetic time series. Genetics.

[15] Iranmehr A, Akbari A, Schlötterer C, Bafna V (2017). CLEAR: Composition of likelihoods for evolve and resequence experiments. Genetics.

[16] Schlötterer C, Kofler R, Versace E, Tobler R, Franssen SU (2014). Combining experimental evolution with next-generation sequencing: a powerful tool to study adaptation from standing genetic variation. Heredity.

[17] Topa H, Jónás Á, Kofler R, Kosiol C, Honkela A (2015). Gaussian process test for high-throughput sequencing time series: application to experimental evolution. Bioinformatics.

[18] Topa H, Honkela A (2016). Analysis of differential splicing suggests different modes of short-term splicing regulation. Bioinformatics.

[19] R Core Team. R: A Language and Environment for Statistical Computing. R Foundation for Statistical Computing, Vienna, Austria. 2018. https://www.R-project.org/.

[20] Li B, Dewey CN (2011). RSEM: accurate transcript quantification from RNA-Seq data with or without a reference genome. BMC Bioinformatics.

[21] Katz Y, Wang ET, Airoldi EM, Burge CB (2010). Analysis and design of RNA sequencing experiments for identifying isoform regulation. Nat Methods.

[22] Turro E, Su S-Y, Gonçalves Â, Coin LJM, Richardson S, Lewin A (2011). Haplotype and isoform specific expression estimation using multi-mapping RNA-seq reads. Genome Biol.

[23] Glaus P, Honkela A, Rattray M (2012). Identifying differentially expressed transcripts from RNA-seq data with biological variation. Bioinformatics.

[24] Bray NL, Pimentel H, Melsted P, Pachter L (2016). Near-optimal probabilistic RNA-seq quantification. Nat Biotechnol.

[25] Bansal V (2010). A statistical method for the detection of variants from next-generation resequencing of DNA pools. Bioinformatics.

[26] Kofler R, Orozco-terWengel P, De Maio N, Pandey RV, Nolte V, Futschik A, Kosiol C, Schlötterer C (2011). PoPoolation: A toolbox for population genetic analysis of next generation sequencing data from pooled individuals. PLoS ONE.

[27] Rasmussen CE, Williams CKI (2006). Gaussian Processes for Machine Learning.

[28] Topa H, Honkela A (2015). Gaussian process modelling of multiple short time series. Proceedings of ESANN 2015, 23rd European Symposium on Artificial Neural Networks, Computational Intelligence and Machine Learning, Bruges (Belgium).

[29] Kass RE, Raftery AE (1995). Bayes factors. J Am Stat Assoc.

[30] Matikainen M-P, Honkela A. GitHub repository of tigreBrowser. https://github.com/PROBIC/tigreBrowser. Accessed 5 Sep 2018.

[31] Honkela A, Gao P, Ropponen J, Rattray M, Lawrence ND (2011). tigre: Transcription factor inference through gaussian process reconstruction of expression for bioconductor. Bioinformatics.

[32] Honkela A, Girardot C, Gustafson EH, Liu Y-H, Furlong EEM, Lawrence ND, Rattray M (2010). Model-based method for transcription factor target identification with limited data. Proc Natl Acad Sci USA.

[33] Honkela A, Peltonen J, Topa H, Charapitsa I, Matarese F, Grote K, Stunnenberg HG, Reid G, Lawrence ND, Rattray M (2015). Genome-wide modeling of transcription kinetics reveals patterns of RNA production delays. Proc Natl Acad Sci USA.

[34] Spies D, Renz PF, Beyer TA, Ciaudo C (2017). Comparative analysis of differential gene expression tools for RNA sequencing time course data. Brief Bioinform.

[35] Illingworth CJR, Mustonen V (2011). Distinguishing driver and passenger mutations in an evolutionary history categorized by interference. Genetics.

[36] Terhorst J, Schlötterer C, Song YS (2015). Multi-locus analysis of genomic time series data from experimental evolution. PLoS Genet.

